# A Study of Caregiver Burden Among Informal Dementia Caregivers in Assisted Living

**DOI:** 10.1093/geroni/igaf122.703

**Published:** 2025-12-31

**Authors:** Molly Perkins, Candace Kemp, Kenneth Hepburn, Regina Shih, Regine Haardoerfer, Carolyn Clevenger, Miranda Moore, Alexis Bender

**Affiliations:** Emory University School of Medicine, Atlanta, Georgia, United States; Georgia State University, Atlanta, Georgia, United States; Emory University, Atlanta, Georgia, United States; Emory University Rollins School of Public Health, Atlanta, Georgia, United States; Emory University, Atlanta, Georgia, United States; Emory University, Atlanta, Georgia, United States; Emory University School of Medicine, Atlanta, Georgia, United States; Emory University, Atlanta, Georgia, United States

## Abstract

Informal caregivers, especially relatives and friends, play a critical role in caring for assisted living (AL) residents with dementia. Limited empirical evidence suggests that caregiver burden may be greater for informal caregivers in AL compared with other long-term care settings, particularly within context of caring for residents with advanced dementia. This study reports baseline findings from a 5-year NIA-funded (RF1AG069114/R01AG069114) longitudinal project designed to address this knowledge gap and identify potential interventions aimed at reducing challenges and improving care and care experiences. Data derived from baseline surveys, including qualitative open-ended questions, with 134 informal caregivers from 39 diverse AL communities in Georgia. We calculated descriptive statistics to characterize the sample and used thematic analysis to analyze the qualitative data. Most participants (mean age = 61) were female (85%), Non-Hispanic White (84%), and had some college education or higher (91%). Close to half (45%) reported mild to moderate burden on the Zarit Burden Interview (ZBI-12). More than one-third (35%) reported severe burden. Despite high levels of education, the average percentage of correct answers on the Dementia Knowledge Assessment Tool (DKAT2) was less than half (47%). In addition to lack of knowledge about dementia and the disease process, qualitative data showed that other key factors contributing to caregiver burden included limited time for self-care, staff turnover and lack of continuity of care, and inconsistent communication from AL staff and hospice. Findings have important implications for interventions aimed at improving care for AL residents with advanced dementia and reducing informal caregiver burden.

